# Supplementary material for: An empirical appraisal of eLife’s assessment vocabulary
Source: PLoS Biol. 2024 Aug 22;22(8):e3002645. doi: 10.1371/journal.pbio.3002645 (PMC11340897; doi:10.1371/journal.pbio.3002645)

### **SUPPLEMENTARY INFORMATION 1: Example stimuli and attention check**

This section contains example stimuli and the attention check for illustrative purposes. The veridical stimuli are available on the Open Science Framework (<https://osf.io/jpgxe/>).

**Supplementary Figure A**. An example summary statement referring to a study’s significance and the corresponding response scale with an arbitrary response shown.


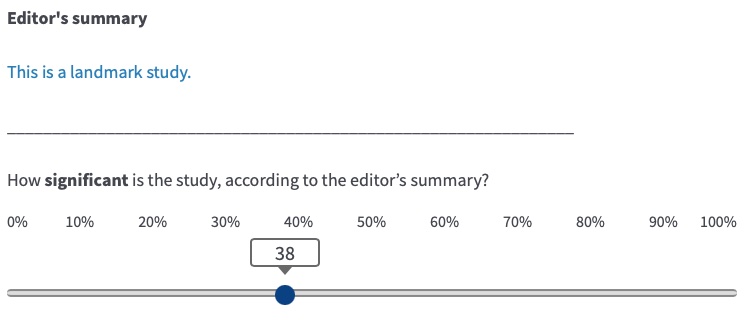


**Supplementary Figure B**. An example summary statement referring to a study’s importance and the corresponding response scale with an arbitrary response shown.


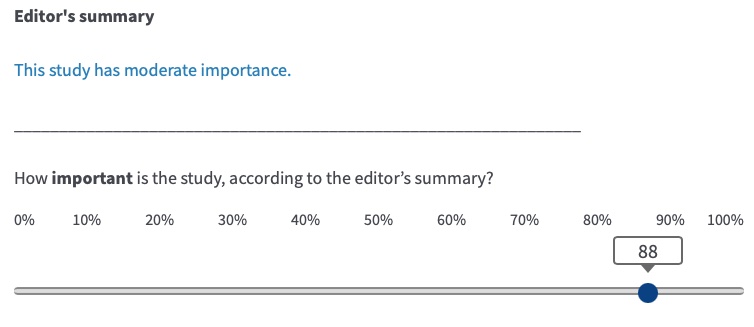


**Supplementary Figure C**. Attention check statement.


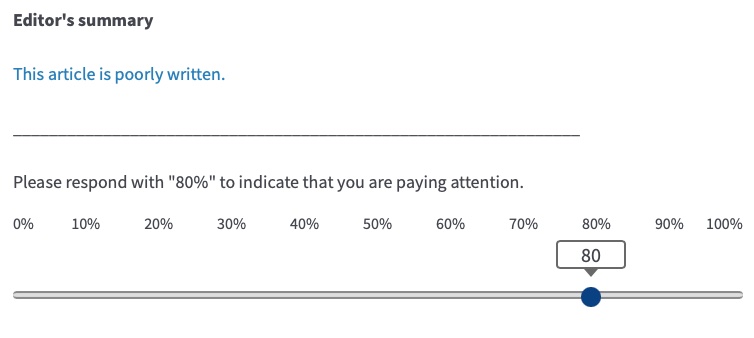

Supplement: S1 Text — (DOCX) [file pbio.3002645.s001.docx]
