# Supplementary material for: An empirical appraisal of eLife’s assessment vocabulary
Source: PLoS Biol. 2024 Aug 22;22(8):e3002645. doi: 10.1371/journal.pbio.3002645 (PMC11340897; doi:10.1371/journal.pbio.3002645)
Supplement: S2 Text — (DOCX) [file pbio.3002645.s002.docx]

### **SUPPLEMENTARY INFORMATION 2: Sample size planning**

We firstly decided that 300 participants was a reasonable sample size target given our resources. We then evaluated the expected statistical power and precision in a plausible scenario, assuming a sample size of 300 and a two-sided test with alpha = .05. For shorthand, we refer to a ‘correct response’ where the observed ranking matches the intended ranking.

In a scenario where 10% of people respond correctly to the eLife vocabulary and incorrectly to the alternative vocabulary, and 20% of people respond correctly to the alternative vocabulary and incorrectly to the *eLife* vocabulary, this would yield a McNemar odds ratio of 2, 95% confidence intervals [1.3-3.1] and statistical power of 0.87 with an exact McNemar test. Analysis code documenting these calculations is available at https://osf.io/8v9n5 (under the heading “Sample size planning”).
