# Supplementary material for: An empirical appraisal of eLife’s assessment vocabulary
Source: PLoS Biol. 2024 Aug 22;22(8):e3002645. doi: 10.1371/journal.pbio.3002645 (PMC11340897; doi:10.1371/journal.pbio.3002645)
Supplement: S3 Text — (DOCX) [file pbio.3002645.s003.docx]

### **SUPPLEMENTARY INFORMATION 3: Task instructions**

This section contains copies of the task instructions for illustrative purposes. The veridical task instructions are available on the Open Science Framework (<https://osf.io/jpgxe/>).

Before starting the study, participants will be presented with the instructions shown in Supplementary Box A and have the opportunity to respond to a practice statement. At the start of each block they will be shown the instructions shown in Supplementary Box B.

**Supplementary Box A**. Pre-study instructions.

| PAGE 1  Thank you for your participation in this study. Please ensure you are in a quiet, distraction-free environment before starting the task. Please give the task your full attention, it will only take about 10 minutes of your time.  PAGE 2  Imagine that you visit the website of a scientific journal to read some articles reporting scientific studies. You see that each article is accompanied by a short summary statement expressing the editor’s opinion of the study report in the article.  We are going to show you 22 statements describing the editor’s opinion of 22 different research studies. For each statement, we’d like you to tell us what you think about a particular aspect of the study using a slider on a scale ranging from 0 to 100%.  After each statement, you will complete a 15 second task involving simple multiplication questions.  PAGE 3  Here’s an example before we start. The editor’s summary appears in blue. In this example, your task is to rate how **clearly written** you think the article is based on the editor’s summary statement. You can click and drag the slider to choose your response.  Practice using the slider now and then click next when you are ready to start the study. If you are having technical problems or anything is unclear, please contact tom.hardwicke@unimelb.edu.au |
| --- |


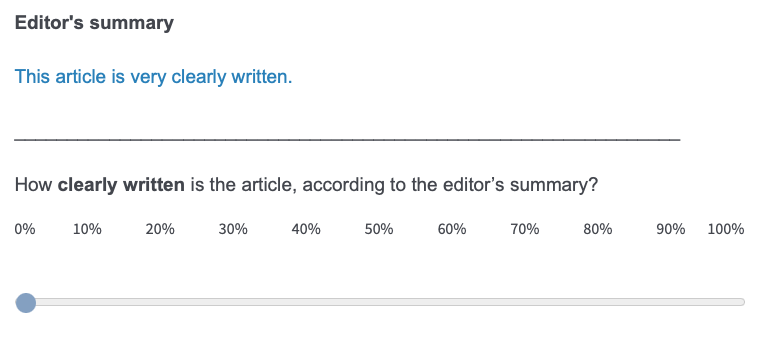


**Supplementary Box B**. Pre-block instructions.

| STRENGTH OF SUPPORT BLOCKS  You will now see statements about the **strength of support** offered by 5/6 different research studies. These statements represent the journal editor’s opinion about the **strength of support** each study offers towards its main claims.  SIGNIFICANCE BLOCK  You will now see statements about the **significance** of 5 different research studies. These statements represent the journal editor’s opinion about the **significance** of each of the study’s main claims.  IMPORTANCE BLOCK  You will now see statements about the **importance** of 5 different research studies. These statements represent the journal editor’s opinion about the **importance** of each of the study’s main claims. |
| --- |
