# Supplementary material for: An empirical appraisal of eLife’s assessment vocabulary
Source: PLoS Biol. 2024 Aug 22;22(8):e3002645. doi: 10.1371/journal.pbio.3002645 (PMC11340897; doi:10.1371/journal.pbio.3002645)
Supplement: S4 Text — (DOCX) [file pbio.3002645.s004.docx]

**SUPPLEMENTARY INFORMATION 4. Peer Community in Registered Reports Design Template.**

This study was originally peer reviewed and received in-principle acceptance from Peer Community in Registered Reports. That platform required the Design Template below. After submitting the Stage 2 manuscript to PLOS Biology, we were asked to add the additional “summary of outcomes” column that summarizes the outcomes for each research aim.

**Supplementary Table A.** Peer Community in Registered Reports Design Template.

| Question | Hypothesis | Sampling plan | Analysis Plan | Rationale for test sensitivity | Interpretation given different outcomes | Theory that could be shown wrong by the outcomes | Summary of outcomes |
| --- | --- | --- | --- | --- | --- | --- | --- |
| Research Aim 1: To what extent do people share similar interpretations of phrases used to describe scientific research? | N/A | See Main Text — sub-section “Sample” and Supplementary Information B. | See Main Text — Analyses for Research Aim 1. | N/A. No hypotheses. | Descriptive. | N/A (applied research question) | See Figures 1 and 2. See Tables 3 and 4. |
| Research Aim 2: To what extent do people’s (implicit) ranking of phrases used to describe scientific research align with (a) each other; and (b) with the intended ranking? | N/A for (a). For (b), we hypothesize that the alternative vocabulary will have higher ranking accuracy than the eLife vocabulary. | See Main Text — sub-section “Sample” and Supplementary Information B. | See Main Text — Analyses for Research Aim 2. | See Supplementary Information B | Mostly descriptive. For the hypothesis test, a significant result will indicate that ranking accuracy is higher for one vocabulary relative to the other, and it would be surprising to observe this difference if the null hypothesis were correct. A non-significant result will imply that the observed results are not surprising under the null hypothesis, and we therefore do have grounds to conclude that the ranking accuracy differs between the two vocabularies. | N/A (applied research question) | See Figures 3, 4, and 5.  On the significance/importance dimension, 59 (20% [15% to 24%]) participants’ implied rankings of the eLife vocabulary aligned with the intended ranking and 188 (62% [57% to 68%]) participants’ implied rankings of the alternative vocabulary aligned with the intended ranking. A McNemar test indicated that observing a difference between the vocabularies this large, or larger, is unlikely if the null hypothesis were true (odds ratio = 8.17, 95% CI [5.11,13.69], p = 1.34e-26).  On the strength of support dimension, 45 (15% [11% to 20%]) participants’ ratings of the eLife phrases were in accordance with the intended ranking relative to 201 (67% [62% to 72%]) participants who correctly ranked the alternative vocabulary. A McNemar test indicated that observing a difference between the vocabularies this large, or larger, is unlikely if the null hypothesis were true (odds ratio = 11.4, 95% CI [6.89, 20.01], p = 5.73e-35). |
| Research Aim 3: To what extent do different phrases used to describe scientific research elicit overlapping interpretations and do those interpretations imply broad coverage of the underlying measurement scale? | N/A | See Main Text — sub-section “Sample” and Supplementary Information B. | See Main Text — Analyses for Research Aim 3. | N/A. No hypotheses. | Descriptive. | N/A (applied research question) | See Figures 1 and 2. See Tables 3 and 4. |
