# Supplementary material for: An empirical appraisal of eLife’s assessment vocabulary
Source: PLoS Biol. 2024 Aug 22;22(8):e3002645. doi: 10.1371/journal.pbio.3002645 (PMC11340897; doi:10.1371/journal.pbio.3002645)
Supplement: S5 Text — (DOCX) [file pbio.3002645.s005.docx]

**SUPPLEMENTARY INFORMATION 5. Expanded versions of Table 3 and Table 4 percentile estimates with confidence intervals.**

| **Supplementary Table A**: Percentile estimates for participant responses to phrases on the significance/importance dimension for eLife and alternative vocabularies. 95% confidence intervals bootstrapped with the percentile method are shown in the square brackets. IQR: Interquartile range. | | | | |
| --- | --- | --- | --- | --- |
| **Importance dimension** | | | | |
| **phrase** | **median** | **IQR** | **25th percentile** | **75th percentile** |
| **eLife vocabulary** | | | | |
| landmark | 94 [92,95] | 12 [8,14] | 86 [84,90] | 98 [96,99] |
| fundamental | 83 [81,85] | 20 [16,22] | 70 [68,74] | 90 [90,91] |
| important | 72 [71,75] | 14 [13,16] | 66 [65,68] | 80 [80,81] |
| valuable | 70 [70,71] | 20 [16,20] | 60 [60,62] | 80 [76,80] |
| useful | 60 [60,63] | 20 [18,20] | 50 [50,52] | 70 [69,70] |
| **Alternative vocabulary** | | | | |
| very high importance | 90 [90,91] | 10 [8,13] | 84 [81,85] | 94 [93,95] |
| high importance | 80 [80,81] | 13 [12,16] | 75 [72,75] | 88 [86,90] |
| moderate importance | 50 [50,51] | 10 [6,13] | 48 [45,50] | 58 [55,60] |
| low importance | 15 [13,18] | 10 [10,12] | 10 [9,10] | 20 [20,22] |
| very low importance | 8 [6,9] | 7 [5,8] | 4 [3,5] | 11 [10,13] |
|  |  |  |  |  |

| **Supplementary Table B**: Percentile estimates for participant responses to phrases on the support dimension for eLife and alternative vocabularies. 95% confidence intervals bootstrapped with the percentile method are shown in the square brackets. IQR: Interquartile range. | | | | |
| --- | --- | --- | --- | --- |
| **Support dimension** | | | | |
| **phrase** | **median** | **IQR** | **25th percentile** | **75th percentile** |
| **eLife vocabulary** | | | | |
| exceptional | 95 [94,95] | 8 [7,10] | 90 [90,90] | 98 [97,100] |
| compelling | 80 [76,80] | 16 [14,19] | 70 [70,71] | 86 [85,90] |
| convincing | 75 [71,75] | 16 [14,20] | 65 [63,67] | 81 [80,85] |
| solid | 74 [71,75] | 17 [15,18] | 65 [64,67] | 82 [80,83] |
| incomplete | 20 [16,20] | 20 [19,22] | 10 [8,10] | 30 [28,30] |
| inadequate | 10 [10,13] | 15 [13,16] | 5 [4,5] | 20 [18,21] |
| **Alternative vocabulary** | | | | |
| very strong support | 88 [85,90] | 12 [10,14] | 80 [80,82] | 92 [91,94] |
| strong support | 77 [75,80] | 15 [11,15] | 70 [70,71] | 85 [81,85] |
| moderate support | 50 [50,51] | 10 [7,13] | 46 [45,49] | 56 [55,59] |
| weak support | 15 [14,18] | 12 [10,15] | 10 [8,10] | 22 [20,25] |
| very weak support | 9 [7,10] | 8 [5,10] | 5 [4,5] | 13 [10,14] |
