# Supplementary material for: An empirical appraisal of eLife’s assessment vocabulary
Source: PLoS Biol. 2024 Aug 22;22(8):e3002645. doi: 10.1371/journal.pbio.3002645 (PMC11340897; doi:10.1371/journal.pbio.3002645)
Supplement: S6 Text — (DOCX) [file pbio.3002645.s006.docx]

**SUPPLEMENTARY INFORMATION 6. Frequency of implied rankings.**

| **Supplementary Table A.** Implied rankings **for the *eLife* vocabulary, significance/importance dimension.** | | |
| --- | --- | --- |
| **Implied ranking** | **n** | **% [CI]** |
| 1-2-3-4-5 | 59 | 20 [15, 24] |
| 1-3-2-4-5 | 32 | 11 [6, 15] |
| 1-2-3-5-4 | 20 | 7 [2, 11] |
| 2-1-3-4-5 | 19 | 6 [2, 11] |
| 1-4-3-2-5 | 15 | 5 [1, 10] |
| 1-3-2-5-4 | 14 | 5 [0, 9] |
| 1-2-4-3-5 | 13 | 4 [0, 9] |
| 2-3-4-1-5 | 13 | 4 [0, 9] |
| 1-4-2-3-5 | 10 | 3 [0, 8] |
| 2-4-3-1-5 | 9 | 3 [0, 8] |
| 3-2-1-4-5 | 9 | 3 [0, 8] |
| 2-3-1-4-5 | 8 | 3 [0, 7] |
| 2-1-4-3-5 | 6 | 2 [0, 7] |
| **Table note**: 42 other rankings with n < 6 are not shown here. CI: Confidence interval. | | |

| **Supplementary Table B**. Implied rankings **for the eLife vocabulary, support dimension.** | | | |
| --- | --- | --- | --- |
| **Implied ranking** | **n** | **% [CI]** |  |
| 1-2-3-4-5-6 | 45 | 15 [11, 20] |  |
| 1-2-4-3-5-6 | 44 | 15 [10, 19] |  |
| 1-2-3-5-4-6 | 29 | 10 [5, 15] |  |
| 2-1-4-3-5-6 | 24 | 8 [4, 13] |  |
| 1-2-5-3-4-6 | 22 | 7 [3, 12] |  |
| 1-2-5-4-3-6 | 20 | 7 [2, 12] |  |
| 2-1-5-3-4-6 | 15 | 5 [1, 10] |  |
| 1-2-4-5-3-6 | 14 | 5 [0, 10] |  |
| 2-1-3-4-5-6 | 14 | 5 [0, 10] |  |
| 1-2-3-4-6-5 | 11 | 4 [0, 9] |  |
| 2-1-5-4-3-6 | 11 | 4 [0, 9] |  |
| 23 other rankings with n < 6 are not shown here. CI: Confidence interval. | | |  |

| **Supplementary Table C**. Implied rankings **for the alternative vocabulary, significance/importance dimension.** | | |
| --- | --- | --- |
| **Implied ranking** | **n** | **% [CI]** |
| 1-2-3-4-5 | 188 | 62 [57, 68] |
| 2-1-3-4-5 | 50 | 17 [11, 22] |
| 1-2-3-5-4 | 35 | 12 [6, 17] |
| 2-1-3-5-4 | 23 | 8 [2, 13] |
| Table note: 4 other rankings with n < 6 are not shown here. CI: Confidence interval. | | |

|  | | |  |
| --- | --- | --- | --- |
| **Supplementary Table D**. Implied rankings **for the alternative vocabulary, significance/importance dimension.** | | |  |
| **Implied ranking** | **n** | **% [CI]** | |
| 1-2-3-4-5 | 201 | 67 [62, 72] | |
| 2-1-3-4-5 | 49 | 16 [11, 22] | |
| 1-2-3-5-4 | 24 | 8 [3, 13] | |
| 2-1-3-5-4 | 20 | 7 [2, 12] | |
| Table note: 6 other rankings with n < 6 are not shown here. CI: Confidence interval. | | |  |
