# Supplementary material for: An empirical appraisal of eLife’s assessment vocabulary
Source: PLoS Biol. 2024 Aug 22;22(8):e3002645. doi: 10.1371/journal.pbio.3002645 (PMC11340897; doi:10.1371/journal.pbio.3002645)
Supplement: S7 Text — (DOCX) [file pbio.3002645.s007.docx]

**SUPPLEMENTARY INFORMATION 7. Further explanation of Kendall’s distance.**

Consider an example case where two individuals are asked to rank three fruits in their favourite order and we want to compare the similarity of the rankings. The individuals provide the following rankings:

Person A = Orange, Apple, Pear

Person B = Pear, Apple, Orange

In this case, Kendall’s distance (K_d_) = 3, because three adjacent pairwise swaps are required to convert Person A’s list into Person B’s list (specifically, we need to swap Apple-Pear, then Pear-Orange, then Apple-Orange.). In this example the maximum K_d_ is 3, so these two rankings are maximally dissimilar.

For our study, we can use K_d_ the measure the similarity between a given participant’s ranking and the intended ranking. For example, we could compare:

Intended (eLife) ranking = useful, valuable, important, fundamental, landmark

(A hypothetical) observed ranking = useful, important, valuable, fundamental, landmark

In this case, K_d_ = 1, because only a single adjacent pairwise swap (valuable <-> important) is necessary to convert the participant’s ranking into the intended ranking.
