# Supplementary material for: An empirical appraisal of eLife’s assessment vocabulary
Source: PLoS Biol. 2024 Aug 22;22(8):e3002645. doi: 10.1371/journal.pbio.3002645 (PMC11340897; doi:10.1371/journal.pbio.3002645)
Supplement: S8 Text — (DOCX) [file pbio.3002645.s008.docx]

**SUPPLEMENTARY INFORMATION 8**. **Demographics of Prolific members.**

Complete demographic information about Prolific members is not available as demographic screening questions are voluntary. Based on the available responses at the time of our study, 30% of Prolific members said they were aged between 18-25, 58% said they were aged between 26-50, and 12% said they were aged between 51-100; asked about their gender, 35% identified as a man, 46% identified as a woman, and 2% identified as non-binary; 18% said they were currently a student; 87% said they were fluent English speakers; 31% said they have UK nationality and 31% said they have USA nationality; when asked to report the highest level of education they have completed, 25% said undergraduate degree, 12% said graduate degree, and 2% responded doctorate degree.
