# Supplementary material for: An empirical appraisal of eLife’s assessment vocabulary
Source: PLoS Biol. 2024 Aug 22;22(8):e3002645. doi: 10.1371/journal.pbio.3002645 (PMC11340897; doi:10.1371/journal.pbio.3002645)
Supplement: Attachment — Submitted filename: Response to reviews.docx [file pbio.3002645.s009.docx]

**Response to reviews**

Manuscript title: Finding the right words to evaluate research: An empirical appraisal of eLife’s assessment vocabulary.

We are grateful to the editor and reviewers for their rigorous assessment of the manuscript and thoughtful comments. Below we provide a point-by-point response to the reviews and note any corresponding changes we have made to the manuscript. All changes are also highlighted in the latest version of the manuscript using ‘track changes’. We have numbered editor/reviewer comments sequentially. Reviewer comments appear in black and our responses are in blue.

**Editor comments**

1) Please add a column to the right of the study design table that reports the actual outcome per row and whether it confirmed or disconfirmed the hypothesis.

**Author Response:** We have added a column as requested.

2) As you will see, both R1 and R3 remain uncomfortable about your level of engagement with eLife, uncomfortable enough to raise this independently, because they did not have a chance to see a revised stage 1. Although it is not a precondition for publication, we would tend to agree and encourage you to engage with eLife about their intention behind the assessment system, as this would make the discussion more straightforward and certain (eg about intentionality). In addition, this work is likely to receive a fair amount of scrutiny post-publication, including the peer-review file, and so the reviewer qualms might be discussed in post-publication commentary around the article.

**Author Response:** We have been in contact with eLife on several occasions, received a positive response, and never received any indication that the vocabulary was not intended to have an ordinal structure. As noted in our cover letter to PCI-RR, while planning the study, we wrote to Damian Pattinson (Executive Director of eLife) and Michael Eisen (then Editor-in-Chief of eLife) and shared a study protocol with them (5th May 2023). The first author (Tom Hardwicke) then met with Damian in person on 8th May 2023 at a conference dinner in Washington D.C. and discussed the proposed study. Finally, we forwarded the Stage 2 manuscript (after it was released as a preprint) to Damian on 8th May 2024 and received a brief, positive response. On the basis of these conversations, we felt reassured that the vocabulary is intended to have an ordinal structure. We don’t mention these conversations in the manuscript because they were informal and therefore we presume off-the-record. The clearest public articulation of eLife’s rationale appears in their article introducing the vocabulary (available at <https://tinyurl.com/3y9y9rs4>) and we cite this in our manuscript.

3) Reviewer 2 also speaks of additional analyses that could be done with the data. Again, this would not be required for publication, but we did want to give you the opportunity to address this report if you wanted. Especially the assessment of whether the frequency of actual ratings might be justified given pre-selection based on quality, or whether the reviewers disproportionately use words on the lower end of the scale, appears to be relatively doable in a reasonable time frame, should you wish to complement the work in that way.

**Author Response:** We are grateful for the suggestion and agree it might be interesting to follow up in future work. We are satisfied that the current study already provides a useful contribution without extending its scope.

4) Could the COI statement for author Simine Vazire be modified in our submission system to state the following?

"SV is a member of the board of directors of The Public Library of Science (PLOS). This role has in no way influenced the outcome or development of this work or the peer-review process, nor does it alter our adherence to PLOS Biology policies on sharing data and materials."

**Author Response:** Yes, we have modified the text as requested.

**Reviewer #1 Comments**

1. I re-read the stage 1 protocol submission, which is registered here: <https://doi.org/10.17605/OSF.IO/MKBTP> (which I will hereafter refer to simply as S1)

The rationale and the stated hypotheses of this Stage 2 manuscript submitted to PLOS Biology (which I will hereafter refer to simply as S2), are indeed the same as approved in S1. The wording between the S1 and S2 is not precisely the same.

A brief digression / sidenote: not a fault of the authors, but I must remark it is a little unhelpful to provide reviewers only PDF files when asking peer reviewers to check the similarity between two separate documents with separate formatting (one with line numbering, one without!). I converted the S1 and S2 into plain text files, chopping-off the S2 at the appropriate places to ensure maximum comparability with the content of the S1. From here a simple bag of words analysis comparing between the plain text of S1 and S2 revealed to me a few linguistic changes that seem to have occurred between S1 and S2 e.g.

In S1: "eLife recently stated that these assessments will use phrases drawn from a common vocabulary…"

In S2: "eLife stated that these assessments would use phrases drawn from a common vocabulary…"

Although there are small changes of wording detectable between the S1 and S2 I did not perceive them to have changed the meaning of the introduction, rationale, or stated hypotheses.

**Author Response:** We can confirm that there are a few minor word changes between S1 and S2 — these are to ensure clarity and accuracy (for example, when we wrote the S1 protocol the word ‘recently’ was accurate, but it seems less accurate now).

2. I note that the authors aimed for a target sample size of 300 in the stage 1 protocol and report a target sample size of 301 in the stage 2 manuscript. This tiny difference is noted by the authors in the stage 2 manuscript and is NOT a significant deviation from the stage 1 protocol.

As far as I can tell the authors have adhered to the approved S1 experimental procedures. The data are to an extent able to test the authors proposed hypotheses.

**Author Response:** We can confirm that this was the only deviation from the S1 protocol.

3. As I noted in my review of S1 I still think it is a bit of a cop-out by the authors to choose not to ask eLife for the broad (e.g. country-level) demographic characteristics of eLife readers - most well-run journals absolutely do hold data on the geography of where in-bound page views are coming from, at least to the country-level if not in much finer detail via standard web traffic analysis captured by e.g. Google Analytics or Matomo. I can see from inspection of their HTML that eLife use Google Analytics to track website usage data. Their global site tag is currently "GTM-WVM8KG" - you'll see it tagged in every single page. They will know the IP address of every visitor to their site and from the IP address will be able to determine the geolocation of the reader with a high degree of accuracy (relatively few readers take steps to obfuscate the IP address they are reading from).

**Author Response:** Thank you — future research may be able to use this information to explore the impact of geographical diversity on vocabulary perception.

4. The conclusions of S2 are valid given the data. However, the conclusions do pre-suppose that eLife intend their vocabulary to have a clear and unambiguous ranking order between words. What if the apparent ambiguity that the data & research has uncovered was _intentional_ on eLife's part? It's not clear to me if the authors here have ever asked eLife if they intended all the words in the vocabulary to have a strict and clear order of ranking. Might the authors want to ask eLife about this? If the intention was to have a clear and unambiguous ranking, why wouldn't eLife have just used numbers rather than words (which clearly as this study and many others show, can be interpreted differently by different people)? Some of the wording in the S2 rather assumes a certain intentionality on behalf of eLife and I'm not 100% convinced that the authors here can speak confidently of eLife's intentionality without directly asking eLife.

**Author Response:** See response to Editor Comment #2.

5. I have examined the data deposited at OSF (<https://doi.org/10.17605/OSF.IO/MW2Q4>) to support S2. The data is in a public repository, it is appropriately described, rights-waived under CC0 1.0 to enable maximum re-use potential, and it is time-stamped (April 6, 2024) to demonstrate that the data was collected after the S1 approval. The data complies with the PLOS data availability policy.

**Author Response:** We appreciate this data availability check.

6. Conclusion: Whilst I retain and reiterate my quibbles from the S1 review stage, this S2 does represent sound, pre-registered research and should be published.

**Author Response:** We’re grateful to the reviewer for their careful scrutiny of the manuscript and helpful comments.

**Reviewer #2 Comments**

7. The second stage of the registered report follows the plan from the first stage. I did not notice any deviations. The results are clearly described and the text in discussion and conclusion is justified given the results.

My biggest regret for the study is that it only purports to show that the eLife vocabulary is not optimal, that the alternative vocabulary is better, but it does not purports to show that the alternative vocabulary is good itself. This is correctly described in the conclusion, where the authors note that future research could find optimal vocabulary, so I do not think the description needs to be changed. I just hope that the alternative vocabulary will not be adopted based only on the results of this study. As the authors correctly note, the wording of the alternative vocabulary overlaps (at the two most extreme points on each end of the scale), which suggests that a different wording might be better.

**Author Response:** We agree that further research will be useful to clarify the utility of the alternative vocabulary (or new vocabularies). As we note (Figure 6), it is possible that changing the format of presentation, rather than the vocabulary itself, might be the most impactful way to improve communication.

8. An informative and useful vocabulary should take into account not only whether the descriptions are correctly ordered, but also how well they cover the importance and support in actual studies. This would be possible to quantify, but the authors opted not do that during the first stage of the registered report. Relatedly, the authors note in the discussion that the eLife vocabulary covers only the upper half of the scale. It would be possible to look at the frequency of actual ratings to see whether this might be justified given pre-selection based on quality, or whether the reviewers disproportionately use words on the lower end of the scale.

**Author Response:** See response to Editor Comment #3.

**Reviewer #3 Comments**

9. Thank you for inviting me to review the Stage 2 of this manuscript. I was excited about the plan at Stage 1 and it's indeed interesting to read the results at Stage 2. First, as far as my assessment goes, the authors have followed the plan carefully, the materials and data are shared, the solitary hypotheses was successfully tested, and the paper thus deserves to be accepted as well as further debated in post-publication discussion. For editors, I acknowledge to have reviewed the html script but didn't run the code in R and am (still) not qualified to comment on the details of Kendall's tau distance.

**Author Response:** We thank the reviewer for their close scrutiny of the manuscript.

10. When reading the final version of the paper in reflection with the Stage 1 review, I must re-address a few points that were raised earlier. Because there was no re-assessment for the revised Stage 1 plan after revisions, I did not have an earlier opportunity to comment on the final Stage 1. Therefore, my Stage 2 review revisits some issues of Stage 1; this is mainly for the open review record and may not entail action from authors. To be clear, these points were considered resolved by the editors/recommenders, so the authors should not be demanded to resolve them but optionally engage if and as they best see fit.

One of my and other reviewers' comments at Stage 1 was to further involve eLife in the study. Considering that the paper systematically uses words like "intended" as the core epistemic lens through which the data are analysed, it still feels awkward that "intentions" have not been backed up beyond the journal's public statement "…to help convey the views of the editor and the reviewers in a clear and consistent manner." Reading author responses post hoc, I can see there has been an informal dinner with one editor before the study, but this was only to inform them about the study. Of course it could be that the short description on eLife website represents all there is to the intended purpose, but that remains unknown for readers at least. I'd suggest having a representative of eLife review the full Stage 2 in order to ensure that their intentions have not been misinterpreted. E.g., it feels strange to publish sentences like "It is unclear if the exclusion of negative phrases was a deliberate choice on the part of eLife's leadership" when it all it takes to figure that out is ask. Moreover, it would've been valuable to hear what specifically eLife means by "consistency" that is operationalised in a specific way here.

**Author Response:** See response to Editor Comment #2.

11. Related to this, while we still don't know what eLife fully intended, their stated decision to use "common vocabulary" implies that the idea was indeed not to use overly quantifying ranking (Likert scale agreement, numeric scores, etc.) but in their own words "rich" vocabulary that perhaps serves to balance between ranking and the inherent vagueness of scientific assessment where comparisons between studies rarely make sense. One could reasonably argue that the results of the study support such balance: eLife vocabulary appears to be somewhat successful at communicating correctly ordered rankings, while not expressing overly confidence on the quantifiability of such ranking. In this regard, it's possible that e.g. reporting percentages (proposed on p. 24) would move the assessment system even more far away from eLife's intended epistemology and/or values. The authors are aware of this, but due to lacking involvement of eLife, all this remains speculation for readers (which could be easily cleared).

**Author Response:** See response to Editor Comment #2.

12. As commented at Stage 1, the paper has one explicit registered test for a hypothesis, which remains difficult for readers to separate from the rest of analytic context on page 17: "The McNemar test indicated that observing a difference between the vocabularies this large, or larger, is unlikely if the null hypothesis were true (odds ratio = 8.17, 95% CI [5.11,13.69], p = 1.34e-26)." The authors must be commended for their generally careful use of language in communicating the finding in the discussion and conclusions. Alas, when I look back at the final Stage 1, it seems that there has been no discussion about the meanings of inference for different effect sizes beyond alpha. Although we can now post hoc agree that OR=8 is plenty, for the record, I am curious what weight the authors put on that effect and why it matters (what would change in inference and discussion had it been OR=2 or OR= 4?).

**Author Response:** The effect sizes can be interpreted directly — an OR of 8.17 indicates that the odds of participants’ implied rankings aligning with the intended ranking were 8.17 times higher for the alternative vocabulary relative to eLife vocabulary. The hypothetical effect sizes mentioned by the reviewer can be interpreted in the same manner (i.e., 2 times higher odds or 4 times higher odds).

13. On page 25 there are limitations: "we did not address whether editor/reviewer opinions provide (a) valid assessments of studies; or (b) whether the vocabularies provide valid measurements of those opinions…" As the authors note later, these are not limitations but different RQs.

**Author Response:** Both of these points clarify the scope of the study and we therefore think it reasonable to refer to them as limitations.

14. I recommend replacing those with limitations, such as a) eLife's intended communication strategy relied on short public text,

**Author Response:** This seems to be a point about eLife’s communications strategy rather than a limitation of our study.

15. b) the decision to skip negative terms for importance/significance might communicate better nuance if indeed non-useful studies are successfully desk rejected (the hypothetical of reviewers disagreeing with editors remains an open empirical question),

**Author Response:** In our view this issue is also not a limitation of the current study and we think it is adequately addressed by footnote one.

16. c) the original vocabulary includes definitions that try to capture certain meanings but (unless I've missed it) the alternative vocabulary does not

**Author Response:** This issue is already addressed in the discussion: “It is unclear if the inclusion of text from the intended phrase definitions will help to disambiguate the phrases and future research could explore this.“

17. and d) the alternative wordings may communicate too much confidence on the quantifiability of scientific work, as in reality such assessment heavily relies on viewpoint differences between individual editors/reviewers, i.e. partial luck, and may thus be suboptimal to quantify with higher precision.

**Author Response:** This issue is already addressed in the discussion:

“...editors may be concerned that using numbers masks vague subjective evaluations in a veil of objectivity and precision. To some extent we share these concerns, however the goal here is not to develop an ‘objective’ measurement of research quality, but to have practical guidelines that improve accuracy of communication. Specifying a numerical range may help to calibrate the interpretations of evaluators and readers, so that the uncertainty can be accurately conveyed.”

18. Because it remains unclear what the optimal number of assessment ranks is (three, four, five, six?), it might be worth suggesting it as a topic for future study, i.e. what would be a sufficient number of ranks that allows journals to distinguish meaningful differences without suggesting too much (artificial) precision.

**Author Response:** Thank you for this suggestion. We have added “Future research could also explore the relationship between the number of items included in the vocabulary and the level of precision that reviewers/editors wish to communicate.” …

19. The last comment goes directly to editors: considering that handing journal, PLOS Biology, is technically a competing journal in life sciences with eLife, it might be suitable to add a COI statement regarding the Stage 2 editorial process.

**Author Response:** This comment is not for us.

In sum, this is an informative study that adds useful knowledge about the discrepancies in how people interpret eLife's chosen terms, and how those discrepancies compare to wordings that are more quantifiable. These data will be helpful especially if and when more journals move toward the eLife model and wish to assess the pros and cons of different wordings in their ranking strategies. I look much forward to post-publication discussions and follow-up journal actions, and wish the best of health to all authors.

Veli-Matti Karhulahti

**Author Response:** We’re grateful to the reviewer for the detailed review and thoughtful comments.
